# Development of a Trivalent Seasonal Influenza Vaccine Using Long α‐Helix‐Mediated Trimeric HA1 Proteins Produced in Baculovirus‐Insect Cell System

**DOI:** 10.1002/mco2.70880

**Published:** 2026-07-19

**Authors:** Ning Guo, Zhibin Xu, Xinyu Chen, Xiaonan Han, Ting Xue, Jinghua Yan, Qingrui Huang

**Affiliations:** ^1^ School of Life Science Anhui Agricultural University Hefei China; ^2^ Changping Laboratory Beijing China

1

Dear Editor:

Seasonal influenza causes approximately 290,000–650,000 deaths annually worldwide, with H1N1, H3N2, B/Yamagata, and B/Victoria lineage viruses responsible for most human epidemics [1]. Current egg‐based vaccines demonstrate highly variable efficacy (10%–60%) due to antigenic mismatch and egg‐adaptation mutations [2]. The baculovirus expression system offers rapid production (<2 months) and has demonstrated safety through approved vaccines, including Flublok [2]. As the immunodominant subunit of the influenza virus hemagglutinin (HA) protein, HA1 is essential for inducing neutralizing antibodies and plays a central role in the virus's immunogenicity [2, 3]. However, recombinant HA1 domains cannot spontaneously trimerize, reducing immunogenicity due to loss of conformational epitopes [4]. Here, we report a trivalent seasonal influenza vaccine comprising trimeric HA1 proteins mediated by the long α‐helix (LAH) domain from HA2 without foreign tags, which elicits superior neutralizing antibody responses and provides complete protection against lethal viral challenge in mice.

Based on the WHO recommendations for the 2023–2024 seasonal influenza vaccine composition, we selected HA1 from three virus strains as the antigenic components for our trivalent seasonal influenza recombinant protein vaccine: A/Wisconsin/67/2022 (H1N1), A/Darwin/6/2021 (H3N2), and B/Austria/1359417/2021 (B/Victoria lineage). We constructed HA1 trimers by fusing the HA1 domain with the conserved LAH region from HA2, which naturally forms stable homotrimers (Figure 1A). Protein constructs incorporated a secretion signal peptide at the N‐terminus and an 8×His tag at the C‐terminus to facilitate purification. The constructs were cloned into baculovirus expression vectors for production using the baculovirus‐insect cell expression system. Recombinant baculoviruses were amplified in insect cell cultures, enabling laboratory‐scale production of these soluble influenza trimer proteins.

**FIGURE 1:**
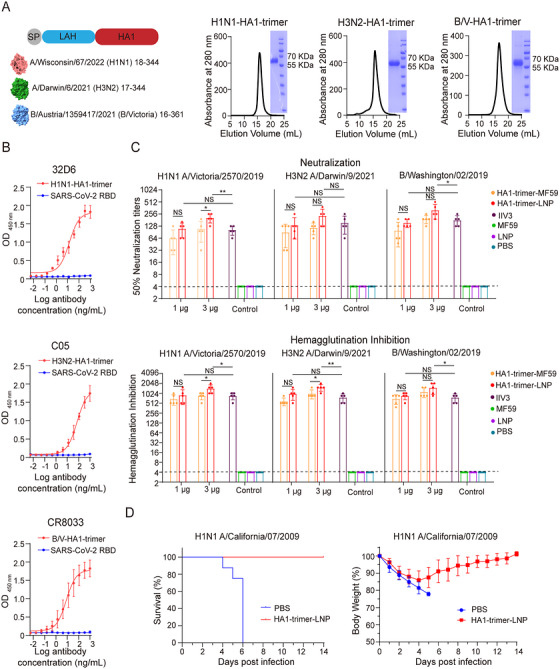
Construction, immunogenicity, and protective efficacy of trivalent HA1‐trimer influenza vaccine candidates. (A) Schematic representation and size exclusion chromatography (SEC) profiles of the HA1 trimer constructs. Left: The constructs were designed based on three seasonal influenza strains—A/Wisconsin/67/2022 (H1N1), A/Darwin/6/2021 (H3N2), and B/Austria/1359417/2021 (B/Victoria)—each comprising a signal peptide (SP), the long α‐helix (LAH) trimerization domain, and the HA1 ectodomain (residues 18–344, 17–344, and 16–361, respectively). Right: SEC chromatograms of the purified trimer constructs. (B) Binding enzyme‐linked immunosorbent assay (ELISA) of HA1‐trimer proteins with strain‐specific monoclonal antibodies. SARS‐CoV‐2 receptor‐binding domain (RBD) protein was used as a negative control. Elution profiles showing absorbance at 280 nm versus volume for H1N1‐HA1‐trimer, H3N2‐HA1‐trimer, and B/V‐HA1‐trimer purified using a Superdex 200 Increase 10/300 GL column. (C) Microneutralization assay and hemagglutination inhibition (HI) titers at 35 days post‐boost. Sera from immunized BALB/c mice were tested for 50% neutralization titers (NT_50_) and HI titers against A/Victoria/2570/2019 (H1N1), A/Cambodia/e0826360/2020 (H3N2), and B/Washington/02/2019. A trivalent inactivated influenza vaccine (IIV3) was used as a positive control. Dotted lines indicate the limit of detection (1:8). (D) Survival and body weight changes following viral challenge. Phosphate‐buffered saline (PBS) control and HA1‐trimer‐lipid nanoparticles (LNP) vaccinated groups were monitored for percent survival and body weight change relative to baseline over 14 days post‐infection. Mice were euthanized when body weight loss exceeded 25% of baseline. For (B–D), data are presented as mean values ± SEM. *p*‐Values were analyzed via one‐way analysis of variance (ANOVA) and Tukey's multiple comparison test (NS, *p* > 0.05; *p < 0.05; **p < 0.01).

Protein characterization by sodium dodecyl sulfate–polyacrylamide gel electrophoresis (SDS‐PAGE) and size exclusion chromatography demonstrated high purity for all three recombinant proteins (Figure 1A). Size exclusion chromatography demonstrated single, homogeneous elution peaks for all three constructs, and SDS‐PAGE analysis confirmed consistent molecular weight profiles across the purified proteins. The monomeric subunits of all three HA1‐trimer variants displayed molecular weights greater than their theoretical values (45 kDa), suggesting glycosylation modifications. Analytical ultracentrifugation revealed molecular weights of 158, 150, and 178 kDa for the three HA1‐trimer proteins, respectively, confirming successful formation of the intended trimeric structures for vaccine formulations. To assess the folding and antigenicity of the recombinant proteins, we performed binding enzyme‐linked immunosorbent assay (ELISA) using strain‐specific neutralizing antibodies: 32D6 for H1N1, C05 for H3N2, and CR80433 for B/Victoria. All antibodies exhibited strong binding to their respective targets, confirming proper conformation and preserved antigenic epitopes of the HA1‐trimer constructs (Figure 1B). Collectively, these data confirm that all three HA1‐trimer antigens were correctly assembled, properly folded, and suitable for downstream immunization studies.

To evaluate vaccine immunogenicity, BALB/c mice (*n* = 5 per group) received HA1‐trimer proteins formulated with either MF59 squalene adjuvant or blank lipid nanoparticles (LNP) (see Materials and Methods, Supporting Information). A trivalent inactivated influenza vaccine (IIV3), containing HA sequences identical to the expressed trimeric HA1 proteins, served as a positive control, while phosphate‐buffered saline (PBS) and adjuvant‐only groups served as negative controls. Following primary immunization, mice received a booster dose three weeks later. Functional antibody responses were evaluated using hemagglutination inhibition (HAI) and microneutralization assays against WHO‐recommended 2020–2021 Northern Hemisphere vaccine strains (see Materials and Methods, Supporting Information). Viral infectivity was standardized to 100 TCID_50_ per well, with neutralization endpoints determined by ELISA‐based viral nucleoprotein detection. Both HA1‐trimer formulations significantly outperformed conventional IIV3 in neutralizing antibody induction (Figure 1C). HA1‐trimer‐LNP consistently demonstrated superior performance over MF59‐adjuvanted formulations (Figure 1C). Control groups showed no neutralizing activity, confirming antigen‐specific responses. HA1‐trimer‐LNP achieved microneutralization titers of 206, 230, and 320 against H1N1, H3N2, and Victoria strains, respectively, representing 2.0‐, 1.5‐, and 1.8‐fold improvements over IIV3 (102, 154, and 179) (Figure 1C). HA1‐trimer‐MF59 generated titers of 109, 116, and 192 against the same strains (Figure 1C). HAI assays corroborated these findings. HA1‐trimer‐LNP produced HAI titers of 1434, 1356, and 1503 HAU against the three vaccine strains—representing 1.7‐, 1.8‐, and 2.0‐fold increases over IIV3 (854, 753, and 753 HAU) and 1.4‐1.7‐fold improvements over HA1‐trimer‐MF59 (855, 944, and 1129 HAU) (Figure 1C). Overall, these immunogenicity data demonstrate that the HA1‐trimer‐LNP formulation elicited the strongest functional antibody responses across all three vaccine‐matched strains.

Based on the superior HAI and virus neutralization activities observed with LNP‐adjuvanted formulations, we selected the 3 µg HA1‐trimer‐LNP group for lethal challenge studies. BALB/c mice (6–8 weeks old, *n* = 8 per group) received prime immunization followed by boost immunization 21 days later. Control mice received PBS using the same immunization schedule. Fourteen days post‐boost, mice were challenged intranasally with 1 × 10^5^ TCID_50_ of A/California/07/2009 (CA07) H1N1 influenza virus. The CA07 strain was selected as the challenge virus due to its high pathogenicity and lethality in BALB/c mice.

Following the viral challenge, mice were monitored daily for 14 days to assess survival rates and body weight changes. PBS control mice exhibited rapid weight loss beginning on day 2 post‐challenge. Progressive mortality was observed in PBS‐treated mice beginning at day 4 post‐infection, with all control animals either succumbing to infection or meeting euthanasia criteria by day 6 (Figure 1D). In contrast, the HA1‐trimer‐LNP vaccinated group demonstrated complete protection against lethal challenge, with 100% survival throughout the 14‐day monitoring period (Figure 1D). Although vaccinated mice experienced mild, transient weight loss reaching its lowest point on day 4 post‐challenge, they recovered progressively and had regained baseline body weights by day 8 (Figure 1D). The observed pattern of initial weight loss followed by recovery in vaccinated mice was consistent with the genetic divergence between the vaccine strain (2023‐2024 WHO‐recommended H1N1) and the challenge virus CA07, which shares approximately 92% homology in the HA gene sequence. Despite this antigenic mismatch, the HA1‐trimer‐LNP vaccine provided complete protection against mortality while allowing for rapid viral clearance and clinical recovery. Overall, these challenge results indicate robust in vivo cross‐protection and effective disease control by HA1‐trimer‐LNP immunization.

In summary, our study demonstrates that LAH‐mediated trimeric HA1 vaccines produced in baculovirus‐insect cells elicit neutralizing antibody responses approximately 2‐fold higher than conventional vaccines when formulated with LNP adjuvant. The superior immunogenicity achieved with LNP adjuvants reflects their intrinsic adjuvant properties that promote robust T follicular helper cell and germinal center B cell responses [5]. Physical mixing of pre‐formed empty LNPs with recombinant proteins offers distinct advantages over encapsulation or chemical conjugation by avoiding complex modification steps that may compromise antigen integrity. This approach also enables independent quality control of individual components prior to formulation, ensuring batch‐to‐batch consistency. Unlike Flublok's full‐length membrane‐bound HA proteins [2], our secreted soluble trimeric HA1 antigens simplify downstream purification while maintaining native conformation, enhancing both scalability and cost‐effectiveness. Notably, our vaccines conferred complete protection against lethal challenge despite antigenic mismatch, demonstrating substantial cross‐protective capacity. The rapid production timeline (<2 months) and manufacturing flexibility position this platform to address annual antigenic drift effectively. Collectively, this baculovirus‐based platform combining LAH‐trimerized HA1 with LNP adjuvants represents a promising next‐generation approach for seasonal influenza vaccination, particularly valuable for pandemic preparedness where manufacturing speed and scalability are critical.

We acknowledge several limitations of this study. First, lethal challenge experiments were conducted only with H1N1, as H3N2 and B/Victoria strains lack intrinsic pathogenicity in standard BALB/c mice. Second, immune responses in mice may not fully recapitulate human immunity, particularly regarding pre‐existing influenza exposure, which is nearly universal in adults but absent in naïve laboratory mice. This pre‐existing immunity could either enhance (through immune recall) or diminish (through original antigenic sin) vaccine responses in humans. Additionally, the optimal antigen dose and adjuvant formulation may differ substantially between mice and humans due to differences in body weight, immune system maturation, and innate immune receptor expression. Future studies comparing alternative trimerization tags, non‐adjuvanted groups, and preclinical models with prior influenza exposure will be important for advancing this vaccine platform toward clinical translation.

## Author Contributions

N.G. and Q.H. designed the study; Z.X. and X.C. performed the experiments. Q.H., X.H., T.X., and J.Y. analyzed and interpreted the data. Q.H. wrote the original manuscript. Q.H. and J.Y. discussed and edited the final manuscript. All authors have read and approved the final manuscript.

## Ethics Statement

All animal experiments were approved by the Committee on the Ethics of Animal Experiments of the Institute of Microbiology, Chinese Academy of Sciences (approval No. APIMCAS2024169). All procedures were carried out in strict accordance with the recommendations outlined in the Guide for the Care and Use of Laboratory Animals.

## Conflicts of Interest

The authors declare no conflicts of interest.

## Supporting information




**Supporting Information**: mco270880‐Supp‐0001‐SuppMatt.docx

## Data Availability

All of the data supporting the findings of this study are available within the text and are available from the corresponding author upon reasonable request.
