# Supplementary material for: Development of a Trivalent Seasonal Influenza Vaccine Using Long α‐Helix‐Mediated Trimeric HA1 Proteins Produced in Baculovirus‐Insect Cell System
Source: MedComm (2020). 2026 Jul 19;7(8):e70880. doi: 10.1002/mco2.70880 (PMC13382368; doi:10.1002/mco2.70880)
Supplement: Supplementary file 1 — Supporting Information: mco270880‐Supp‐0001‐SuppMatt.docx [file MCO2-7-e70880-s001.docx]

**Supporting information**

**Development of a Trivalent Seasonal Influenza Vaccine Using Long α-Helix-Mediated Trimeric HA1 Proteins Produced in Baculovirus-Insect Cell System**

**Affiliations**

**Authors:**

Ning Guo^1†^, Zhibin Xu^1,2†^, Xinyu Chen^2^, Xiaonan Han^2^, Ting Xue^1^, Jinghua Yan^1✉^ , Qingrui Huang^1✉^

**Affiliations:**

^1^School of Life Science, Anhui Agricultural University, Hefei, China

^2^Changping Laboratory, Beijing, China.

^†^These authors contributed equally to this work.

^✉^Corresponding: [huangqr@cpl.ac.cn](mailto:huangqr@cpl.ac.cn) (Q.H.), [yanjh@cpl.ac.cn](mailto:yanjh@cpl.ac.cn) (J.Y.)

**Materials and Methods**

**Cell lines and virus strains**

SF9 cells (B82501, Gibco) were cultured in Sf-900 III SFM medium at 27°C. HEK293T cells (ATCC, CRL-3216) and Madin-Darby canine kidney (MDCK) cells (ATCC, CCL-34) were maintained in Dulbecco's modified Eagle's medium (DMEM) supplemented with 10% fetal bovine serum (FBS) at 37°C in a 5% CO₂ atmosphere. Chicken red blood cells (1%, CF1037, G-lone) were used for hemagglutination inhibition assays.

Influenza virus strains H1N1 A/California/07/2009, H1N1 A/Victoria/2570/2019, H3N2 A/Darwin/9/2021, and B/Austria/1359417/2021 were propagated in specific pathogen-free (SPF) embryonated chicken eggs according to standard protocols.

**Gene construction and cloning**

HA1 trimeric constructs were designed by fusing the HA1 domain with the long α-helix (LAH) region from HA2 to promote stable homotrimer formation. The constructs included: H1N1-HA1 trimer: HA protein residues 403-474 and 18-344 (GenBank accession: OQ203982.1); H3N2-HA1 trimer: HA protein residues 403-474 and 17-344 (GenBank accession: PX230021.1); B/Victoria-HA1 trimer: HA protein residues 403-474 and 16-361 (GenBank accession: PV725666.1).

All constructs incorporated an N-terminal secretion signal peptide and a C-terminal 8×His tag. Monoclonal antibodies 32D6, C05, and CR8033 were constructed by linking variable regions to mouse IgG1 constant regions.

**Protein expression and purification**

Recombinant proteins were expressed in SF9 insect cells infected with recombinant baculoviruses at a multiplicity of infection (MOI) of 5, with viral titers exceeding 1×10⁹/mL. Proteins were initially purified using HisTrap HP affinity chromatography columns or HiTrap Protein A affinity columns, followed by size exclusion chromatography using Superdex 200 columns (GE Healthcare). Protein purity and molecular weight were assessed by SDS-polyacrylamide gel electrophoresis (SDS-PAGE).

**Antigen-Antibody Binding Assay**

ELISA plates (Costar 96-well) were coated with 200 ng HA1 trimeric proteins per well in 50 mM carbonate-bicarbonate buffer (pH 9.6) overnight at 4°C. Strain-specific antibodies (32D6, C05 VPGSGW, and CR8033) were serially diluted 3-fold and incubated for 1 h at 37°C. After washing, HRP-conjugated anti-mouse Fc antibody (Yeasen) was added. Reactions were developed with 50 μL of 3,3′,5,5′-tetramethylbenzidine (Beyotime Biotechnology) and stopped with 50 μL of 2 M sulfuric acid. Absorbance was measured at 450 nm using a microplate reader (PerkinElmer).

**Vaccine formulation**

Blank lipid nanoparticles (LNPs) were prepared using a microfluidic mixing system. The lipid formulation consisted of an ionizable cationic lipid, phosphatidylcholine, cholesterol, and PEG-lipid at a molar ratio of 50:10:38.5:1.5. The total lipid concentration in the ethanol phase was 6 mg/ml. The LNPs were synthesized via microfluidic mixing with a citrate buffer (aqueous phase) flow rate of 9 mL/min and an ethanol phase flow rate of 3 mL/min (flow rate ratio = 3:1). After synthesis, the LNPs were subjected to ultrafiltration for buffer exchange into PBS (pH 7.4) and stored at 4°C until use.

For mouse immunization, antigen proteins were diluted to specified concentrations (0.02 mg/mL per antigen for low dose or 0.06 mg/mL per antigen for high dose) in PBS and mixed with equal volumes of MF59 squalene adjuvant (AVT) or blank LNP by vortex mixing. Each mouse received 100 μL vaccine via intramuscular injection.

**Microneutralization assay**

Neutralization assays were performed in BSL-2 facilities. MDCK cells were seeded at 15,000 cells per well in 96-well plates with DMEM containing 1% FBS. Heat-inactivated mouse sera were serially diluted and mixed with H1N1 A/California/07/2009 virus at 100 TCID₅₀ per well final concentration. The mixture was transferred to MDCK cells and incubated at 37°C with 5% CO₂ for 72 h. Positive controls consisted of virus and cell mixtures without serum. Cells were fixed with 80% acetone for 10 min, air-dried, and washed before incubation with rabbit anti-influenza A virus nucleoprotein antibody (Abcam) for 1 h. Following standard ELISA procedures, neutralization titers were defined as the reciprocal of the maximum dilution where OD₄₅₀ values plus negative control values were less than half the difference between positive and negative controls.

**Hemagglutination inhibition (HAI) assay**

Serum samples from immunized mice were treated with receptor-destroying enzyme and serially diluted 2-fold. Diluted sera were mixed with H1N1 A/Victoria/2570/2019, H3N2 A/Darwin/9/2021, or B/Austria/1359417/2021 (4 HA units) and transferred to V-bottom 96-well microtiter plates. 1% chicken red blood cells were added to each well, and plates were incubated at room temperature for 20 min. HAI titers were defined as the reciprocal of the highest serum dilution completely inhibiting hemagglutination.

**Mouse immunization and viral challenge**

Six- to eight-week-old female BALB/c mice were purchased from Beijing Vital River Laboratory Animal Technology Co., Ltd. and housed under specific pathogen-free (SPF) conditions (22 ± 2°C, 12 h light/dark cycle) with free access to food and water. For adjuvant comparison experiments, mice were randomly assigned to groups (n = 5 per group) and received 100 μL of antigen formulated with MF59 (AVT) or blank LNP via intramuscular injection, followed by a booster immunization with the same formulation 3 weeks later. Two weeks post-boost, sera were collected for antibody analysis. For vaccination and challenge experiments, mice were randomly assigned to groups (n = 8 per group) and received 100 μL of HA1-trimer-LNP vaccine via intramuscular injection, followed by a booster 3 weeks later. Two weeks post-boost, mice were challenged intranasally with 1×10⁵ TCID₅₀ of H1N1 A/California/07/2009 (CA07). Survival rates and body weight were monitored daily for 14 days.

**Statistical analysis**

Statistical significance was analyzed using unpaired Student's t-test for two groups and ordinary one-way ANOVA with multiple comparisons for multiple groups. All analyses were performed using GraphPad Prism 8.0 software without data exclusion.
